# Organ donation for research purposes: a qualitative focus group study on the views of donor families, transplant recipients and heart failure patients in the UK

**DOI:** 10.1136/bmjopen-2025-107992

**Published:** 2025-12-23

**Authors:** John Onsy Louca, Nicole Asemota, Johanna Thren, Alex Manara, Sai Bhagra, Lu Wang, Johannes Bargehr, Nigel Burton, Sue Burton, Karen Rockell, Joao Pedro Nuñes, Paul White, Marius Berman, Stephen Pettit, Antonio Rubino, Amir Reyahi, Stephen Large, Sanjay Sinha, Catherine Wilson

**Affiliations:** 1 Cambridge Stem Cell Institute, Cambridge University, Cambridge, UK; 2 Department of Transplantation, Royal Papworth Hospital, Cambridge, England, UK; 3 Department of Surgery, University of Cambridge, Cambridge, Cambridgeshire, UK; 4 Department of Anthropology, University of Durham, Durham, England, UK; 5 Intensive Care Unit, Southmead Hospital, Bristol, UK; 6 Donor Family Network, Bexley, UK; 7 UK Organ Donor Transplant Research Network, Peterborough, UK; 8 Addenbrooke’s Hospital, Cambridge, England, UK; 9 Luton and Dunstable University Hospital NHS Foundation Trust, Luton, England, UK; 10 Department of Pharmacology, University of Cambridge, Cambridge, UK

**Keywords:** Heart failure, Drug Therapy, Transplant surgery

## Abstract

**Abstract:**

**Background:**

Declined donor organs and explanted recipient organs may hold considerable value for biomedical research, particularly in advancing knowledge of disease mechanisms and supporting drug development. However, public perceptions of such use, and preferences for how consent should be obtained, remain underexplored.

**Methods:**

Four workshops were held across the UK to examine the views of organ donor families and transplant recipients regarding the use of human organs in research, with a focus on myocardial regeneration. Each workshop included three brief presentations on transplantation and cardiac regeneration, followed by facilitated small-group discussions. Observational notes were taken to capture participants’ perspectives on the use of organs unsuitable for transplantation. A follow-up survey generated both quantitative and qualitative data, the latter analysed using thematic analysis.

**Results:**

Participants expressed strong support for the use of declined donor and explanted recipient organs in research. Transplant recipients frequently cited a desire to give back to the National Health Service (NHS), while donor families viewed research use as a meaningful way to honour their loved ones when transplantation was not possible.

**Conclusion:**

This exploratory study highlights widespread support for using non-transplantable organs in research among individuals with personal experience of transplantation. The findings suggest a need for further research into how best to support and inform potential donors and families. Participants emphasised the importance of sensitive communication, clear consent processes and transparency regarding the use of donated organs.

**STRENGTHS AND LIMITATIONS OF THIS STUDY**

- The workshops included multiple stakeholder perspectives, facilitating communication between clinicians, clinical and qualitative researchers, recipients, patients and donor families.
- We used a mixed-data collection approach by collecting data before and after the workshops, which provided three separate occasions for participants to share their thoughts and experiences.
- This study was limited by sampling bias due to its sole focus on individuals with prior exposure to transplantation. There is a need to reach a broader range of individuals, covering members of the public who have little prior exposure to the topic and have not been previously impacted by it.
- The data in this study are subject to recall bias, in that they asked participants to recall whether or not they had previously had the opportunity to express a preference regarding donation for research, relying on their recollected experiences.
- There was an attrition of individuals who filled in the presurvey form and who actually attended the workshop. There was further attrition of individuals who filled in the postworkshop survey.

## Introduction

 Deceased organ donation is an altruistic act by the donor and the relatives who consent. A single act of donation can save or improve multiple lives. Deceased donation represents the main source of organs for use in both abdominal and thoracic transplantation, with heart transplantation taking place exclusively using organs from deceased donors. Between 2023 and 2024 in the UK, there were 1510 deceased organ donors, resulting in 237 heart, 140 lung, 854 deceased liver and 2257 deceased kidney transplants.^1^,^5^ In 2020, the consent process for organ donation in England changed following the implementation of the Organ Donation (Deemed Consent) legislation, which presumes that all potential donors who have not formally registered a donation preference on the National Organ Donor Register or expressed an opposition to donation are deemed to have wanted to donate their organs after their death.^6^ When relatives are asked to sign the consent and authorisation form for organ donation in hospital, they also have the opportunity to indicate whether they consent to the use of their relative’s organs for research, should any organ donated no longer be considered suitable for transplantation.

Every year, a significant number of organs are retrieved from deceased donors but are not subsequently transplanted. The existing infrastructure for the use of donated organs that are not transplanted for research has successfully facilitated much liver and kidney research. In contrast, only a minority of hearts which are turned down for transplantation are used in research. A recent paper analysing the use of turned-down organs in research demonstrated that between 2008 and 2023, only 18 hearts out of a possible 6044 donors (ie, 0.3%) were used in research.^7^ One of the reasons for non-use includes unacceptable ischaemic time—the heart can tolerate up to 30 min of warm ischaemia.^8^ Additionally, use of hearts in research is dependent on the availability of a cardiothoracic retrieval team (who do not attend the majority of retrievals). Consequently, in cases where a heart has been declined for use in transplantation but consented for use in research, it is therefore likely not to be used.

Under the current system, organs can be given to the Organ Donation and Transplantation (ODT) research team if informed consent for their use in research has been obtained from the donor’s relatives or nominated representatives. Studies included in the ODT Research Registry are offered these organs under the current system for generic consent, which applies in such cases. Where studies plan to remove ‘untransplantable tissue’ purely for research, the Human Tissue Authority must licence this type of donation, and the specialist nurse team must be trained to facilitate donation consent for (study-) specific donation.^9^ For example, study-specific donation might take place for research aimed at increasing the transplantability of donated organs.^10^ Additionally, there are national organisations such as the UK Organ Donation and Transplantation Research Network, which is an all-organ initiative between clinical, patient, public and scientific representatives and aims to support research in organ donation and transplantation.^11^ The current National Organ Donation System for Research has a heterogeneous infrastructure and comprises multiple different organisations, studies and disciplines and various national and international funders.^10^

The development of clinical-grade ex-situ perfusion machines has enabled the development of donation after circulatory death heart transplant programmes, as well as greatly extended preservation times in liver transplantation.^12^,^14^ In recent years, the repurposing of ex-situ perfusion machines has been used to test novel therapeutics on explanted recipient and deceased donor organs declined from transplantation.^15^ This technology has been used to test the expression of a novel Vascular Endothelial Growth Factor-A messenger Ribonucleic Acid (VEGF-A mRNA) in explanted recipient hearts.^16^

There is a gap in the literature examining perceptions on organ donation for research using explanted organs or organs unsuitable for donation among transplant recipients, deceased-donor relatives and patients with heart failure. Beyond this, there is little information about the views of recipient and donor relatives on regenerative cardiovascular research. However, the academic literature stresses the importance of appropriate dissemination of information on transplant outcomes and organ usage because of its impact on the deceased-donor relatives’ overall donation experience.^17^ To begin the process of addressing this need, a series of workshops were organised for relatives of deceased organ donors, transplant recipients and patients with heart failure.

## Aims

The workshops aimed to understand the views of transplant recipients, deceased-donor relatives and patients with heart failure on the use of organs for research, where such organs were either diseased hearts explanted from transplant recipients at the time of transplant surgery or those found unsuitable for transplant at the time of donation. A secondary aim of the workshops was to understand the view of participants on regenerative cardiovascular research.

## Methods

We carried out mixed methods data collection during four workshops. The workshops were led by clinicians from the Royal Papworth Hospital and academics from the University of Cambridge with an interest in using turned-down organs and explanted recipient organs to test novel therapies for cardiac regeneration.

The study used focus group discussions, participant observations, survey responses and thematic analysis. We explored the anticipated support and communication needs (both at the time of consent and in the future) surrounding organ donation for research among a group with donation- and transplant-related lived experience. Attendees provided their written consent for the use of their anonymised contributions for the purposes of research. The Department of Anthropology at Durham University provided ethical approval (project ID: 2917, review reference: ANTH-2025-2917-4261) for the use of observational and survey data gathered during observation of the workshop’s design and delivery for use in research, following informed consent. To avoid confronting attendees with unexpected discussions about potentially upsetting topics, recruitment focused on individuals with prior knowledge of donation and transplantation processes, lived experience and a willingness to discuss donation and transplantation in the context of death, loss and personal health challenges. During recruitment, individuals were informed that participation in these workshops would enable the research team to gain insight into their views on the use of organs unsuitable for donation in research. Further information on recruitment is available in the online supplemental file 1. Table 1 summarises the content of the workshops. The workshops comprised three presentations, followed by facilitator-led small group and collective group discussions on a range of questions. Facilitators took notes during the small group discussions and were responsible for feeding these back when the whole group reconvened. After the workshop, facilitators shared the information on what their group had discussed.

**Table 1:** Settings, number of attendees and dates of the workshops

	Workshop 1	Workshop 2	Workshop 3	Workshop 4
Location	Gonville & Caius College, Cambridge	Cambridge Stem Cell Institute	NHSBT Clinical Biotechnology Centre, Bristol	Online*
Date	15/02/2024	15/03/2024	24/05/2024	24/09/2024
Time	18:00–20:30	12:30–15:00	12:30–15:00	18:30–20:30
Number of attendees	17	6	12	38†

Summarising the location, date, time and number of attendees at the four workshops.

* The last workshop was held on Zoom to maximise the number of participants attending the workshops without worrying about geographical constraints.

† There were three repeat attendees at this workshop.

NHSBT, NHS Blood & Transplant.

This design for the workshops was chosen to enable effective two-way communication between clinicians/ academics and participants. The small group discussions enabled participants to voice their thoughts in a non-intimidating environment, with the open-group discussion at the end, enabling facilitators to feedback what their group had discussed.

### Workshop overview

There were three introductory talks at the outset of the workshop. The first of these was on transplantation, the second on cardiac regeneration and the last on the potential use of declined donor organs in a manner that complies with the Human Tissue Act (2004), as all organ donation in the UK currently does.^18^ The topics of these talks were chosen to represent areas of research in cardiac regeneration that would benefit from the use of donated human organs. These talks were delivered by subject experts in these areas of research and, as such, were able to explain the research and answer questions from workshop participants. More information on the talks can be found in the online supplemental file 1.

Following the talks, participants were split into small groups with 5–7 participants per group. In workshop 2, small numbers resulted in no further group division. Focus group discussions lasted between 30 min and 45 min. The questions used by the facilitators to prompt discussions can be found in the online supplemental file 1.

Once formed, groups were invited to reflect together before reconvening to discuss their findings. These were shared predominantly by the facilitator of the group, but individuals were also given the opportunity to add additional points when discussing with the larger group. In addition to the observational data gathered during the workshops, survey responses were generated after the workshop. Participants were sent an anonymous electronic questionnaire (boxes1  2) via email and asked to record their views on the use of donor organs unsuitable for transplant and explanted recipient organs for use in research.

Box 1 **Preworkshop questionnaire**

- On a scale of 1–10, with 1 being wholly opposed and 10 being wholly in support, how do you feel about the use of turned-down organs from transplantation being used for research?
- On a scale of 1–10, with 1 being wholly opposed and 10 being wholly in support, how do you feel about the use of organs taken out of recipients receiving a new heart being used for research?
- To use organs from recipients receiving a new heart, we need consent. When would you like us to gain this consent?
- Who do you think is the best person to speak to you about this work? Would you prefer one of the researchers or one of the clinical team to discuss this work with you?
- Currently, there is no national organ retrieval system for research.^1^ Would you support the development of a national organ retrieval service that can use these organs for research to develop new therapies?

Box 1 summarises the questions asked in the preworkshop questionnaire.

Box 2 **Postworkshop questionnaire**

- Did you enjoy the workshop, (1 being not at all, 10 being very much enjoyed)
- We are keen to continue our work engaging with the public on our research. How do you suggest is the best way to do this?
- On a scale of 1–10, with 1 being wholly opposed and 10 being wholly in support, how do you feel about the use of turned-down organs from transplantation being used for research?
- Has this changed as a result of the workshop? If so, how has it changed?
- On a scale of 1–10, with 1 being wholly opposed and 10 being wholly in support, how do you feel about the use of organs taken out of recipients receiving a new heart being used for research?
- Has this changed as a result of the workshop? If so, how has it changed?
- To use organs from recipients receiving a new heart, we need consent. When would you like us to gain this consent?
- Who do you think is the best person to speak to you about this work? Would you prefer one of the researchers or one of the clinical team to discuss this work with you?
- Currently, there is no national organ retrieval system for research. Would you support the development of a national organ retrieval service that can use these organs for research to develop new therapies?
- Who would you recommend this workshop to? (friends, family, any member of the public, etc)
- Was this the kind of information you would like to learn about our research?
- Are there any other topics that you would like to see covered in future workshops?
- Would you be happy to come to future events and talks in the future?
- What do you think is the best way for us to engage with the public about this kind of work?

Box 2 summarises the postworkshop questionnaire, which was designed not only to give attendees the opportunity to change their thoughts but also to understand how their views had changed as a result of the talks.

The questionnaires were used in conjunction with the discussions from the workshops to better understand the thoughts and feelings of the participants. Categorical responses were summarised as counts (percent), and numerical responses were summarised as mean±SD.

### Participant observation during the workshops

The participant observers comprised speakers and the facilitators for small group discussions. They took anonymised field notes during the workshop and observed the focus group discussions that were hosted. Detailed notes akin to transcription of the spoken contributions were produced in the first instance, and any non-anonymised data were held by the data controller for subsequent analysis and collated into a main document. Data were shared by members of the study to ensure fair and accurate interpretation of the results. Thematic analysis was used to analyse the qualitative data generated in observational and interview notes.^19^ Further detail is available in the online supplemental file 1.

## Results

**Figure 1:**
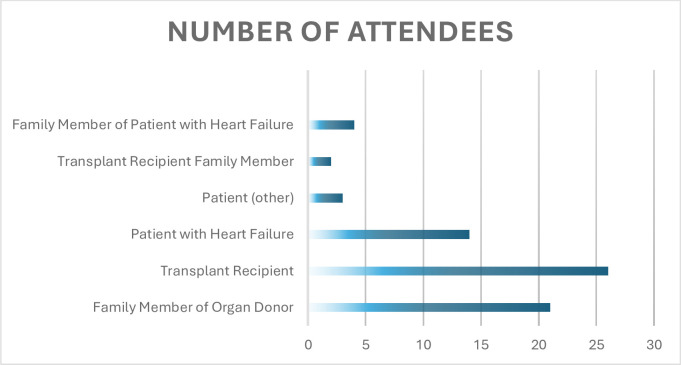
Demographics of the 70 participants attending the workshops.

In total, there were 70 participants across the four workshops. Three participants attended multiple workshops. The backgrounds of the participants are shown in figure 1. Of the 70 attendees, 47 were female, and 23 were male. The results of the postworkshop questionnaire are summarised in table 2.

**Table 2:** Results from postworkshop questionnaires

In support of turned-down donor organs in research (1–10)	9.1±1.4
In support of explanted recipient hearts in research (1–10)	9.4±1.1
Rating of the workshop (1–10)	9.0±1.2
Would you recommend the workshop to others? (Yes)	86.5%
Was this the information you wanted to learn at these events? (Yes)	92.3%
Would you be willing to come to future events? (Yes)	88.5%

The results of the postworkshop questionnaires. Numeric results are calculated as mean±SD. There were 18 participants who did not complete the post workshop questionnaire.

### The conversation regarding use of hearts in research

Transplant recipients agreed that consent for use of explanted organs for research should be ascertained during the consent process for transplantation. The donor family members in attendance had different recollections about the consent process, even though consent to the use of deceased donor organs for research is already embedded into the routine consent and authorisation process for organ donation for transplantation purposes. Attendees felt that consenting for turned-down organs to be used in research should be a distinct part of the conversation when approaching family members of organ donors. Moreover, many attendees at the workshop felt that the choice to donate organs, either clinically or, if this was not possible, in research could be made by the donor during their lifetime. One donor family member stressed, “It helped tremendously knowing that the organs were being used and if needed to help in research then still going to a fantastic cause”—indicating potential positive impact on having the opportunity to donate an organ unsuitable for transplantation for use in research. Additional contribution indicated variation surrounding this view, with some families indicating their reluctance for an organ to be used for research despite their willingness to donate the organ for transplantation. Participants suggested that consent for both research and transplantation should be sought whenever organ donation is discussed with an individual, but that donation for transplantation ‘should always be prioritised’. Attendees felt that the clinical donation team, usually a specialist nurse in organ donation or doctor who looked after the patient (with a pre-existing basis of trust or specialist training), should facilitate the conversation.

### Explanted recipient hearts in research

Among recipients, the risk of a sense of survivor’s guilt emerging following the transplant was highlighted and the notion that this could be somewhat mitigated if the recipient’s own organ could also be donated was surfaced.^20^ Some recipients spoke of the sense of pride they felt for their own organ that had continued to keep them alive and battle on, and that the prospect of the organ having a continued use outside their body was positive. Moreover, there was a desire to give back to society and the healthcare system, after they gave the recipients a second chance at life through transplantation. Participants felt that there was little risk of perceived pressure that could be put on recipients. The recipients stated that they trusted their transplant team enough not to feel pressured. Some recipients shared that they had “assumed my old [organ] would have gone for research” but that when they had asked, they were told “no-one asked that before and as far as they were aware it had not”. Similarly, some recipients expressed that where they did consent to their explanted organ being used for research, they had not received final updates about how the organ had been used. One shared, “*I still have my disease, I want somebody to find a cure […] I donated my [organ] to research – I wish I had hear what happened to it, I regret not knowing something”. Others stressed they would want to know whether the organ had been ultimately cremated*. Many recipients expressed their support for research into treatments for cardiac conditions and believed there would be support for heart donation for research among cardiac patients and interest groups, reflecting after the workshop “it is now clear that research needs these organs to help in the future”. The prospect of being able to support the possible development of better drug treatments or a cure was viewed as highly valuable by many.

### Turned-down donor hearts in research

There was a perceived need to separate the conversation and consideration for organ donation for transplantation and organ donation for research, with one participant stressing the risk of people “getting mixed-up messages, […] the message might come across the wrong way”. These participants felt that the research consideration added an additional level of complexity to what was already a process frequently perceived as lengthy and complex. Among donor relatives, there was confusion regarding the factors that influence communication about the donation outcome following organ donation for transplantation and organ donation for research. The significance of the possibility to donate and the potential positive sense of being able to contribute to the deceased’s ‘legacy’ were highlighted. Some donor family members felt “the ethical case for this is compelling” and that “it helped tremendously knowing that the organs were being used” in cases where the use of explanted organs for transplantation was not possible, describing the use of organs in research as ‘a fantastic cause’.

The need for ‘full and sensitive explanation on the use and storage of the organs retained for research’ to help ‘win hearts and minds’ was stressed by donor families in attendance. They explained, “at the time the family are giving their consent for organ donation it should be remembers that they have just lost their loved one […] the organs that cannot be used are still very much part of the person they have just lost”. While the desire for such information varied, attendees felt there was a risk that researchers could strike a tone that was perceived as upsetting. They were worried information could be “a little bit too scientific – the humanity needs to be involved in that context as well”. Consequently, differing ontologies of the body among healthcare providers and potential organ donors or their families were identified as potential barriers in the communication about organ donation for research. Opportunities to recognise the donation through respectful communication that acknowledged the difficulty of the situation and honorary ceremonies for the donor, such as the Order of St John UK Award for Organ Donation.^21^ Charity support and the help offered by the Donor Family Care Service were highlighted as potential tools to provide reassurance and comfort. Participants indicated they would want to have the option to know the research the organs went on to support and what would happen to the organs and tissues after their use in this research. One attendee who had consented to the use of an organ for research on behalf of a deceased loved one shared, “I would not have felt comfortable with the [organ] just being incinerated. Being given the [organ] back was really important”. Not having clarity about what happened to the organ following the donation contributed to ongoing speculation and confusion for some, indicating a risk of a lack of closure following the loss.

### A formal system for using organs in research

All participants felt that a formalised system for organ donation for research would require the availability of information, later, of what research was supported, appropriately developed communication materials about the research, and ongoing communication with the donors/donor relatives about what had happened to the donated organs. All attendees felt that there was a need to ensure that the conversation about donation for research would need to happen during the consent process for organ donation. Moreover, they mentioned that steps needed to be taken that no pressure was placed on the potential donor or their relatives, and that the clinical team, ideally with special training, would be best placed to facilitate the conversation. Attendees emphasised that there was a need to establish clear boundaries between considerations for organ donation for transplantation and donation for research, with one stressing, “I worry the message might come across in the wrong” which could mean ‘much needed organs get denied to patients who are in need’. Consequently, participants emphasised the need to ensure that any risks of contributing to refusal and pre-empting donation for life-saving care were carefully assessed and prevented. All participants felt they would want to have the option of knowing what research was conducted with the help of the organ they donated. Moreover, they cautioned that a cost-benefit analysis would need to be performed on the justifiability of ‘using an operating theatre just to remove organs for research’ if this was being considered.

Attendees expressed support for the possibility to advance research into novel treatment and prevention approaches for cardiac conditions, stressing that the “benefits […] of research for future patients are exciting” but agreed that further public engagement work with various stakeholders was needed to develop a coordinated system for the use of donated hearts for research. Such work would need to include communication with various religious, socioeconomic, ethnic and cultural subgroups. Lastly, attendees highlighted that the lack of knowledge about organ transplantation and donation among members of the public needed to be addressed and that modern communication channels such as social media or the NHS app could be used for this purpose.

## Discussion

### Proposals on how to further improve the system going forward

The above findings indicate a need to improve the process of donating organs for research purposes in the UK. Further consultative work following this exploratory study would need to be carried out to ensure the process is optimised and works for both healthcare professionals and for donors and their family members going forward.

The main findings from this study were:

- Strong support among attendees for the use of turned-down organs and explanted recipient organs in research. This was often due to an altruistic desire to help others in need and maximise the potential benefits of donation. A need for a more streamlined donation for the research process with greater clarity about when an explanted organ could be used in research was identified.
- A perceived opportunity among organisers to update the NHS organ donor register (ODR) to allow individuals to register their decision regarding organs donated for research should their organs be unsuitable for use in transplantation.
- There was a prevalent sense of a moral responsibility to maximise the utility of donated organs, whether the organ is used clinically or turned down and used for research, with the caveat of care around the language and information used to facilitate the donation.
- A need for clearer communication with family members of organ donors and transplant recipients regarding ongoing research. There was a clear appetite from many participants to have the option to engage further with research and to receive updates about the progress of this research.

One suggestion from the participants was to raise public awareness of the possibility to register on the ODR using the NHS app. There is a need for a widespread awareness of organ donation and nation-wide conversations surrounding this topic, so that family members are aware of their loved ones’ views at the time of organ donation conversations.

### Limitations

There were 78 participants who filled in the preworkshop surveys. 70 individuals attended the workshops, and 51 individuals filled in the postworkshop survey. This may result in selection bias, with participants who filled in the postworkshop survey more likely to have a positive view of the workshop and of organ donation and support the research. Second, the study is subject to sampling bias because individuals who attended the workshop would likely have been more interested in this research than individuals who chose not to attend, and most attendees had transplant-related lived experience or experience with organ failure. The study was subject to recall bias in cases where contributions were based on recollected donation experiences on which statements regarding attitudes and preferences about organ donation for research were based. Further demographic data could have been collected from attendees to enable further insights into the study population. This exploratory study focused on discussing the topic with individuals on a hypothetical basis and indicates an opportunity to address a gap in the literature relating to the experiences of donors and donor families who donate their organs for research. Future work should reach out to members of the general public and people who have successfully consented to the use of organs for research.

### Conclusion

There was clear support for the use of turned-down organs and explanted recipient organs in research among the workshop attendees. The findings indicate a need to improve the consent process and follow-up information process used to facilitate organ donation for research. The findings reported in this paper constitute an initial exploration, and further work is necessary to expand the evidence base and advance knowledge on the procedural, legal and experiential complexities in this area.

## Supplementary material

10.1136/bmjopen-2025-107992 online supplemental file 1

## Data Availability

Data sharing not applicable, as no datasets generated and/or analysed for this study.
