# Supplementary material for: Organ donation for research purposes: a qualitative focus group study on the views of donor families, transplant recipients and heart failure patients in the UK
Source: BMJ Open. 2025 Dec 23;15(12):e107992. doi: 10.1136/bmjopen-2025-107992 (PMC12730744; doi:10.1136/bmjopen-2025-107992)
Supplement: online supplemental file 1 [file bmjopen-15-12-s001.docx]

**Organ donation for research purposes: A qualitative focus group study on the views of donor families, transplant recipients and heart failure patients in the United Kingdom: Supplement**

**Methodology**

*Recruitment*

Potential participants were approached by local and national charities who acted as gatekeepers. These included the Norfolk Zipper Club, the Donor Family Network, the UK Organ Donor & Transplant Research Network, the RPH Heart Transplant Recipient Facebook group and Cardiomyopathy UK. An invitation was distributed via mailing lists from these charities to individuals. Interested individuals who volunteered to attend were advised to contact the team organising the event for further details. At this point individuals were sent a pre-workshop questionnaire and given the details for the meeting.

*Questions used to prompt discussion with individuals during workshops*

- Do you support the use of diseased organs removed during transplantation for research purposes?
- Do you support the use of turned-down organs from transplantation in research?
- This kind of work needs your consent, when would you like us to gain this consent?
- Who do you think is the best person to speak to you about this work?
- When we are asking for your permission to use organs, do you feel the need to know what kind of research we are doing?

*Short Scientific Talks*

The talks given in the workshops were on the following:

1. “The extraordinary gift of a heart, by way of introduction. Reflections of heart transplantation”. This talk was a quick overview of heart transplantation, describing its efficacy and more importantly its shortcomings and hence the need for further research into curing heart failure. This talk was delivered by a consultant cardiac surgeon with a particular interest in organ retrieval.
2. The second talk explored either stem cell or gene therapies as potential avenues to improve heart failure, particularly after myocardial infarction. These were named “Regenerating the heart” or “Mending the broken heart” respectively. These were delivered by academics from the University of Cambridge with an interest in cardiac regeneration using both stem cell and gene therapies.
3. The last talk was centred around the use of ex-situ machine perfusion to test novel therapeutics in explanted recipient hearts and turned down donor hearts. The title of this talk was “Ex-Situ Heart Perfusion & Next Steps in Therapeutic Development”. This talk was delivered by a clinician with an interest in cardiac transplantation and ex-situ organ perfusion.

*Thematic Analysis*

Contributions from the structured focus group discussions and semi-structured large group discussions were thematically analysed using a series of colour-coding systems and mind maps. Recurring themes were identified and subsequent informal reflection with the workshop organisers was used to cross-compare findings that could be used as an additional contrasting perspective for further reflection.
