# Supplementary material for: Organ donation for research purposes: a qualitative focus group study on the views of donor families, transplant recipients and heart failure patients in the UK
Source: BMJ Open. 2025 Dec 23;15(12):e107992. doi: 10.1136/bmjopen-2025-107992 (PMC12730744; doi:10.1136/bmjopen-2025-107992)
Supplement: Supplementary file 2 [file bmjopen-2025-107992.reviewer_comments.pdf]

PEER REVIEW HISTORY

BMJ Open publishes all reviews undertaken for accepted manuscripts. Reviewers are asked to complete a checklist review form and are provided with free text boxes to elaborate on their assessment. These free text comments are reproduced below.

ARTICLE DETAILS

Title (Provisional)

Organ donation for research purposes: A qualitative focus group study on the views of donor families, transplant recipients and heart failure patients in the United Kingdom

Authors

Louca, John Onsy; Asemota, Nicole; Manara, Alex; Thren, Johanna; Large, Stephen; Bhagra, Sai; Wang, Lu; Burton, Nigel; Burton, Sue; Bargehr, Johannes; Rockell, Karen; Nuñes, Joao Pedro; White, Paul; Berman, Marius; Pettit, Stephen; Rubino, Antonio; Reyahi, Amir; Sinha, Sanjay; Wilson, Catherine

---

VERSION 1 - REVIEW

**Reviewer**	**1**
**Name**	**Vail , Emily A**
**Affiliation**	**Hospital of the University of Pennsylvania**
**Date**	**07-Sep-2025**
**COI**	**None**

In this qualitative study, investigators examined the perspectives of relatives of deceased organ donors, patients with heart failure, and transplant recipients (and their relatives) on the use of donated (and explanted) organs for research purposes and, secondarily, research focused on 'heart regeneration'.

The authors used a comprehensive approach, starting with four invited focus group sessions and pre- and post-session surveys, aimed at identifying key areas of importance.

Group participants shared important and actionable insights, including the potential value of a research donation opportunity when transplant opportunities are not available, clarifying the timing and information shared during the research consent process (including pre-registration specific to research), and the need for comprehensive follow-up for donor families and patients regarding research use and final disposition.

The study fills an important knowledge gap, with the following limitations:

- Volunteer bias

- Generalizability to the UK public or similar family and patient groups outside of the UK (both of population demographic differences, as well as donation laws and processes)

- It includes a heterogeneous cohort expected to have variable knowledge and experience of the donation process

I have two additional questions for clarification:

1) The investigators mention that the UK research consent includes consent for interventions delivered before organ recovery. This appears to be otherwise unexplored in the manuscript, but represents a crucial distinction – did study subjects differentiate between this type of research and others described? If not, this was a missed opportunity, for which the description of this specific type of research may be removed from the introduction.

2) Did the investigators specifically elicit stakeholders' perspectives on the use of decedents ineligible for organ donation as whole body research donors?

I believe the manuscript would be strengthened by a few changes:

1) Greater focus. For example, although participants viewed a presentation about 'cardiac regeneration', the investigators did not present questions related to this topic in the focus groups, nor did they elicit participants' views on this topic. Thus, the description could be otherwise removed from the manuscript.

2) Including additional context about the problem studied. Is there a dearth of hearts donated for research, or does the amount recovered meet current needs? How much does family or patient refusal contribute to this problem? Or, do the other factors mentioned (e.g., lack of a recovering thoracic surgical team) contribute more? If the former, then information gathered here is more useful than if there is a broad public willingness to use these organs, but practical factors are the main barrier.

3) Descriptions of study and individual task (e.g., follow-up survey) non-response rates.

4) In Table 4, only the 'yes' proportion needs to be presented (No is implied).

5) Inclusion of key (anonymized) participant quotes illustrating key themes.

6) Inclusion of a COREQ reporting guidelines checklist (https://www.equator-network.org/reporting-guidelines/coreq/).

**Reviewer**	**2**
**Name**	**Stukov, Yuriy**
**Affiliation**	**University of Florida**
**Date**	**09-Sep-2025**
**COI**	**None**

The authors investigated public perceptions of donor families and transplant recipients regarding the use of declined donor organs and explanted recipient organs. This is a thoughtful and timely organized research in an era of increasing amount of patients who require solid organ transplantation. This paper provides valuable insight into problem and has a strong potential to contribute to both scholarly and practical conversations in transplantation ethics.

Congratulations to the research team

VERSION 1 - AUTHOR RESPONSE

Reviewer: 1
Dr. Emily A Vail , Hospital of the University of Pennsylvania
Comments to the Author:
In this qualitative study, investigators examined the perspectives of relatives of deceased organ donors, patients with heart failure, and transplant recipients (and their relatives) on the use of donated (and explanted) organs for research purposes and, secondarily, research focused on 'heart regeneration'.

The authors used a comprehensive approach, starting with four invited focus group sessions and pre- and post-session surveys, aimed at identifying key areas of importance.

Group participants shared important and actionable insights, including the potential value of a research donation opportunity when transplant opportunities are not available, clarifying the timing and information shared during the research consent process (including pre-registration specific to research), and the need for comprehensive follow-up for donor families and patients regarding research use and final disposition.

We thank Dr Vail for her positive comments and for her time and expertise in reviewing this manuscript. We have responded to her comments and made the suggested amendments to the manuscript.

The study fills an important knowledge gap, with the following limitations:
- Volunteer bias
- Generalizability to the UK public or similar family and patient groups outside of the UK (both of population demographic differences, as well as donation laws and processes)
- It includes a heterogeneous cohort expected to have variable knowledge and experience of the donation process

We agree that this piece of work has these limitations. We have now made these limitations clearer in the 5 bullet points after the abstract as requested by the editor. Indeed, this exploratory work is not sufficient to generalise to the whole UK public, but it is the basis for further work that is urgently needed in this area. Moreover, by engaging with stakeholders who have previous experience with the organ donation process we were able to streamline the workshops and did not need to explain the basics of the organ donation process.

I have two additional questions for clarification:
1) The investigators mention that the UK research consent includes consent for interventions delivered before organ recovery. This appears to be otherwise unexplored in the manuscript, but represents a crucial distinction – did study subjects differentiate between this type of research and others described? If not, this was a missed opportunity, for which the description of this specific type of research may be removed from the introduction.

In the UK families are approached for consent to organ donation for transplantation. At the same time families are also consented for research on organs not suitable for transplantation. Consent to organ donation for transplantation does not imply consent to use of organs for research. A family can consent to organ donation for transplantation but not agree to donation of organs for research purposes. Also. antemortem interventions are used to increase the likelihood organ donation and utilisation for transplantation and not to enable the retrieval of organs for research purposes. Consent to organ donation does not assume consent to all antemortem interventions. Instead, the use of antemortem interventions are subject to comprehensive professional guidance as per the Donation Actions Framework (please see - https://nhsbtdbe.blob.core.windows.net/umbraco-assets-corp/27065/donation-actions-framework-v10-june-2022.pdf for further information).

2) Did the investigators specifically elicit stakeholders' perspectives on the use of decedents ineligible for organ donation as whole body research donors?

Currently in the UK a retrieval team is only mobilised to recover organs for transplantation. If the organs cannot be utilised for transplantation, then they may be used for research if the family had previously agreed. There is currently no established national retrieval service for organs or bodies for research purposes. It is evident is that there is already incredibly poor utilisation of hearts from a large pool of individuals who have already consented for organ donation and have already had organs procured. Transplant teams have no involvement with individuals who decide on whole body donation. This is usually coordinated at a local centre level and has no involvement from NHS Blood & Transplant (the governing body of transplantation in the UK).

I believe the manuscript would be strengthened by a few changes:
1) Greater focus. For example, although participants viewed a presentation about 'cardiac regeneration', the investigators did not present questions related to this topic in the focus groups, nor did they elicit participants' views on this topic. Thus, the description could be otherwise removed from the manuscript.

We thank the reviewer for this comment. We discuss the use of donated organs in research with a view on organ (specifically heart regeneration work). We felt these talks were crucial both in the workshop and worth mentioning in the manuscript as this contextualises the reasons behind using turned down donor organs in research. Moreover, as the workshop included transplant recipients and individuals with heart failure, we found that this talk was important and that it was worth providing this in the manuscript.

2) Including additional context about the problem studied. Is there a dearth of hearts donated for research, or does the amount recovered meet current needs? How much does family or patient refusal contribute to this problem? Or, do the other factors mentioned (e.g., lack of a recovering thoracic surgical team) contribute more? If the former, then information gathered here is more useful than if there is a broad public willingness to use these organs, but practical factors are the main barrier.

There is very little utilisation of hearts in research in the UK. There is virtually no utilisation of whole hearts for organ regeneration research. We do not know the exact reasons as to why there is such poor utilisation of hearts. Part of this may be due to logistical issues and part of it due to lack of awareness – both for patients and clinicians/ academics. This exploratory work was done to better understand whether there was public support for use of these turned down organs as to date there has been no literature published on this. This exploratory work will enable further work to better understand the general public's views of this research and will enable engagement with policy makers to push for further utilisation of hearts.

We have now more clearly demonstrated the poor utilisation of hearts. We have cited a paper demonstrating that between 2008-2022, out of 6044 turned down heart donors, only 18 hearts were used in research (i.e. 0.3%). The reasons for this poor utilisation are not fully understood and there is a need for further work both to understand the reasons for this very low utilisation rate and then to subsequently increase it.

3) Descriptions of study and individual task (e.g., follow-up survey) non-response rates.

We thank the reviewer for this comment. We have now expanded on this and described the number of pre-workshop surveys filled in, the number of attendees and the number of post-workshop surveys filled in. We expand on this in the limitations and mention how this is a further weakness with the study. – see lines 242-245.

In addition to this we have provided information in the supplementary on the questions that were asked in the workshop as well as the topics of the talks. We have also included the details of the survey questionnaires before and after the workshops as Tables 2 & 3. We hope that the added details adequately answers the reviewers point.

4) In Table 4, only the 'yes' proportion needs to be presented (No is implied).

Thank you for this comment. We have now amended the table appropriately.

5) Inclusion of key (anonymized) participant quotes illustrating key themes.

We performed thematic analysis on the participant quotes to summarise the key ideas. From the pre-workshop survey, the post-workshop survey and the workshops itself there was a large amount of information. However, we have now included quotes in the relevant parts of the results section to support our arguments.

6) Inclusion of a COREQ reporting guidelines checklist (https://www.equator-network.org/reporting-guidelines/coreq/).

We thank the reviewer for this comment. As per the editors instructions we have filled in an SPQR checklist. We believe that the COREQ reporting guidelines largely have the same function, therefore we have not filled them in.

Reviewer: 2
Dr. Yuriy Stukov, University of Florida
Comments to the Author:

The authors investigated public perceptions of donor families and transplant recipients regarding the use of declined donor organs and explanted recipient organs. This is a thoughtful and timely organized research in an era of increasing amount of patients who require solid organ transplantation. This paper provides valuable insight into problem and has a strong potential to contribute to both scholarly and practical conversations in transplantation ethics.

Congratulations to the research team

We thank Dr Stukov for his kind words and the time he has taken to review this manuscript.

VERSION 2 - REVIEW

**Reviewer**	**1**
**Name**	**Vail , Emily A**
**Affiliation**	**Hospital of the University of Pennsylvania**
**Date**	**04-Dec-2025**
**COI**	

In this revised manuscript, the authors report results of a UK-based qualitative study of donor families and patients with end-stage heart disease (before and after transplant) and their views on the use of donated and explanted organs for biomedical research.

Notable revisions in this version include:

- Updated title to reflect improved focus on biomedical research (and excluding the previously included focus on organ regeneration, which I recommended removing in my original review)

- Expanded introduction describing the scope of the problem

- Addition of clarifying details throughout

- Addition of illustrative quotes from study subjects

These changes substantially strengthen the manuscript. I look forward to its publication.
